# The Impact of Newer Biological Disease Modifying Anti-Rheumatic Drugs on Cardiovascular Risk Factors: A 12-Month Longitudinal Study in Rheumatoid Arthritis Patients Treated with Rituximab, Abatacept and Tociliziumab

**DOI:** 10.1371/journal.pone.0130709

**Published:** 2015-06-26

**Authors:** Sella Aarrestad Provan, Inger Jorid Berg, Hilde Berner Hammer, Alexander Mathiessen, Tore Kristian Kvien, Anne Grete Semb

**Affiliations:** 1 Dept. of Rheumatology, Diakonhjemmet Hospital, Oslo, Norway; 2 Preventive Cardio-Rheuma clinic, Dept. of Rheumatology, Diakonhjemmet Hospital, Oslo, Norway; Institute of Immunology, Rikshospitalet, NORWAY

## Abstract

**Objectives:**

To assess whether treatment with one of three novel biological DMARDs; rituximab, abatacept or tocilizumab reduce cardiovascular disease (CVD) risk factors in patients with rheumatoid arthritis (RA).

**Methods:**

This is an open, observational and prospective study with visits at baseline, 3, 6, and 12 months. Patients were assigned to receive rituximab, abatacept or tocilizumab according to clinical indications assessed by an independent rheumatologist. Disease activity was quantified by the disease activity score (DAS28) and extensive ultrasonography. CVD risk was assessed by total cholesterol (TC), high-density lipoprotein cholesterol (HDL-c), blood pressure and arterial stiffness measurements [pulse wave velocity (PWV) and augmentation index (AIx)]. Within group change in disease activity and CVD risk over 3 months was explored using paired samples bivariate tests. Predictors of change in CVD risk at 3 months were identified in linear regression models. Changes in CVD risk markers over the 12- month follow-up in patients receiving rituximab were assessed by mixed models repeated analyses.

**Results:**

24 patients on rituximab, 5 on abatacept and 7 on tocilizumab were included. At 3 months PWV was significantly reduced in the tocilizumab group only, but at 12 months rituximab patients showed a significant reduction in PWV. Reduced inflammation at 3 months was associated with increased TC and HDL-c in the entire cohort.

**Conclusion:**

Treatment with tocilizumab and rituximab reduces PWV, a marker of CVD risk, in patients with RA.

## Background

Clinical disease activity and systemic inflammation are independent predictors of mortality from cardiovascular disease (CVD) in rheumatoid arthritis (RA) [1], and studies have shown that methotrexate and tumour necrosis factor-α inhibitors (TNF-α) therapy can reduce CVD mortality [2]. Pulse wave velocity (PWV) is the gold standard of arterial stiffness measurements and predicts CVD mortality in several studies [3]. The augmentation index (AIx) is a measure of the augmentation of central pressure and also an independent predictor of CVD mortality [3]. Treatment with TNF-α inhibitors has been shown to improve the level of several CVD risk markers, including PWV and AIx [2–3]. Total cholesterol (TC) and high-density lipoprotein cholesterol (HDL-c) are however paradoxically negatively correlated to level of disease activity [4].

Over the past decade novel immunomodulatory biologics have become available for patients with RA. Rituximab, abatacept and tocilizumab are three biological disease-modifying antirheumatic drugs (DMARD) with proven efficacy in RA [5–7]. We hypothesised that these new biological DMARDS would have positive effects on markers of CVD risk.

The aim of this study was to assess the effect of rituximab, abatacept and tocilizumab on markers of CVD risk in patients with RA.

## Methods

This was an open, observational, longitudinal study. Eligible for inclusion were: Patients fulfilling the American College of Rheumatology (ACR) classification criteria for RA, 18–80 years of age, and designated to receive rituximab, abatacept or tocilizumab after evaluation by a clinical rheumatologist independent of this study. Patients could re-enter the study if they switched to another study drug during the study period. Patients were excluded if unable to participate due to physical or cognitive difficulties, suffering from atrial fibrillations incompatible with pulse wave registrations or when in need of anti-hypertensive medication, and/or statins during the follow-up period.

The Regional Committees for Medical and Health Research Ethics of South-Eastern Norway approved the study and the patients gave a signed informed consent on inclusion.

### Data collection

The patients were examined prior to starting with biological DMARDs and at 3, 6 and 12 months. Participants were requested to abstain from food/drinks and smoking for at least 3 hours prior to examination.

### RA disease activity

A trained study-nurse examined 28 joints and calculated the disease activity score with 3 variables (DAS28) [8]. Ultrasonographic examinations (US) were performed by an experienced sonographer, (HBH) (Siemens Antares, Sonoline, USA) using a 5–13 MHz probe, fixed settings. Thirty-six joints and 4 tendons were scored using standardized projection in B-mode (USBM) (synovial hypertrophy and joint fluid combined) and in power-Doppler (USPD) (presence of vascularization) on a 4-point scale [9]. The US examiner was blinded for results from all previous examinations.

### Biomarkers

Soluble biomarkers were examined consecutively; erythrocyte sedimentation rate (ESR) by the Westergren method, C-reactive protein (CRP), TC, HDL-c, low-density lipoprotein cholesterol (LDL-c) and triglycerides (after a minimum 3-hours fast) by COBAS 6000 (Roche Diagnostics, Basel Switzerland). Atherogenic index was calculated as TC/HDL-c. Brachial blood pressure (BP) was measured after a 5 minute rest in a supine position using the OMRON M7 (Kyoto, Japan). Pulse wave analyses (PWV and AIx) were performed using the Sphygmocor apparatus (Atcor, West Ryde, Australia) as previously described [10].

### Statistics

Bivariate comparisons of change in CVD risk markers at 3 months were made using the Paired samples T-test or the Related Samples Wilcoxon Sign Rank test as appropriate. Predictors of change in CVD risk markers at 3 months were identified in separate linear regressions models. Predicted values, residuals and Cooks distances were plotted in significant models. Change in CVD risk markers and disease activity at 3 months was calculated by subtracting the baseline value from the value at 3-months.

Mixed model linear regression was utilized to examine change in level of CVD risk markers over the 12-month follow-up in the patients receiving rituximab. Time and measures of disease activity (DAS28, USBM and USPD) were entered as possible predictors in models adjusted for age and sex. An unstructured covariance matrix was used in our analysis. The residuals and predicted values were examined for normality in a scatter diagram. All analyses were performed using SPSS version 21. P-values < 0.05 were considered significant.

## Results

Patient demographics are presented in Table 1. Thirty-eight patients were included, 6 were excluded after baseline registration. 4 patients re-entered the study after switching to either tocilizumab or abatacept prior to the 6 month follow-up.

**Table 1:** Baseline demographics.

Variables	Rituximab	Abatacept	Tocilizumab
Participants at 0,3, 6 and 12 months	24,24,20,15	5,5,2,0	7,7,5,3
Age in years (range)	56.9 (25–72)	53.9 (41–67)	52.3 (36–59)
Female gender number (%)	17 (71)	5 (100)	6 (86)
Current smoker number (%)	5 (22)	0 (0)	0 (0)
CVD number (%)	1 (4)	0 (0)	0 (0)
CRP mg/L	9.6 (7.9)	11.2 (14.3)	18.1 (14.2)
ESR mm/hour	29.5 (14.3)	32.6 (22.9)	41.4 (25.9)
TC mmol/L	5.8 (1.2)	5.6 (0.7)	5.8 (1.6)
HDL-c mmol/L LDL-c mmol/L	1.8 (0.5) 3.5(1.0)	1.8 (0.5) 3.3 (0.5)	2.0 (0.6) 3.5 (1.3)
Triglycerides mmol/L	1.3 (0.5)	1.0 (0.4)	1.2 (0.5)
DAS28	4.7 (1.3)	5.1 (0.6)	5.3 (1.2)
USBM	23.1 (14.0)	22.3 (23.4)	34.0 (1.4)
USPD	9.9 (9.6)	8.8 (14.2)	24.0 (4.2)
Systolic BP mmHg	128.0 (16.2)	108.6 (10.7)	133.0 (22.3)
Diastolic BP mmHg	79.4 (8.5)	66.8 (8.1)	84.4 (14.4)
AIx %	25.2 (12.2)	24.7 (15.8)	21.8 (12.6)
PWV m/s	7.7 (1.4)	6.8 (1.2)	7.8 (1.6)

Table presents crude mean values (SD) and number (percentages) for counts unless otherwise specified.

CVD: Cardiovascular disease defined as myocardial infarction or cerebral insult, CRP: C-reactive protein, ESR: Erythrocyte sedimentation rate, TC: Total cholesterol. HDL: High density lipoprotein cholesterol, LDL-c: Low density lipoprotein cholesterol mmol/L, DAS28: Disease activity score 28 joints. USBM: Ultrasonography B-mode score, USPD: Ultrasonography Power doppler score, Systolic BP: Systolic blood pressure, Diastolic BP: Diastolic blood pressure, AIx: Augmentation index, PWV: Pulse wave velocity

Of the 36 patients finally included, 24 received rituximab, 5 abatacept and 7 tocilizumab. Due to low numbers in the abatacept and tocilizumab arms after 3 months, only data from the rituximab patients were analysed over the 12- month period. No adverse events requiring hospital admission occurred during the study period.

In the bivariate analysis of related samples at 3 months, only the patients treated with tocilizumab displayed a significant reduction in CVD risk, measured by PWV. CRP and ESR were also reduced over the 3 months period in the tocilizumab group (Table 2).

**Table 2:** Within group change in CVD risk markers at 3 months.

	Rituximab	p	Abatacept	p	Tocilizumab	p
CRPª n36	5.8 (26.9)	0.77	-1.4 (14.0)	1.00	-17.0 (14.3)	0.02
ESR n35	-2.2 (16.8)	0.54	1.0 (15.0)	0.89	-37.1 (26.2)	0.01
TC n32	0.1 (0.7)	0.74	-0.2 (0.6)	0.40	0.34 (1.0)	0.37
HDL-c n32	-0.1 (0.4)	0.44	0.2 (0.2)	0.16	0.1 (0.4)	0.64
LDL-c n32	-0.1 (0.4)	0.40	-0.2 (0.6)	0.45	0.5 (0.6)	0.20
Triglycerides n32	-0.5 (0.6)	0.71	-0.0 (0.4)	0.96	-0.0 (0.5)	0.97
DAS28 n32	-0.8 (0.9)	0.00	-0.2 (0.9)	0.70	-2.8 (1.4)	0.00
US-BMª n25	0.1 (6.3)	0.85	8.8 (5.7)	0.07	n/a	
US-PDª n25	0.3 (6.0)	0.76	5.0 (3.9)	0.11	n/a	
SBP n35	-1.3 (10.1)	0.53	4.0 (9.6)	0.40	-11.5 (18.6)	0.15
DBP n35	-1.0 (6.1)	0.46	4.2 (6.7)	0.23	-6.7(9.3)	0.10
AIx n34	1.7 (7.5)	0.30	4.3 (6.1)	0.19	2.6 (8.2)	0.44
PWV^a^ n35	0.1 (0.8)	0.69	0.3 (0.6)	0.23	-0.9 (1.0)	0.03

Paired samples T test or Related Samples Wilcoxon Sign Rank test as appropriate.

Mean (Standard deviation) presented. Negative values signify a reduction (ª)Wilcoxon Sign Rank test performed.US scores were not analyzed in the Toc group as 4 of these patients had missing baseline data of this parameter.n number.

N number, CRP: C-reactive protein mg/L, ESR: Erythrocyte sedimentation rate mm/hour, TC: Total cholesterol mmol/L, HDL-c: High density lipoprotein cholesterol mmol/L, LDL-c: Low density lipoprotein cholesterol mmol/L, DAS28: Disease activity score 28 joints, US-BM: Ultrasonography B-mode score US-PD: Ultrasonography Power doppler score, Systolic BP: Systolic blood pressure mmHg, Diastolic BP: Diastolic blood pressure mmHg, Aix: Augmentation index %, PWV: Pulse wave velocity m/s.

In a linear regression model use of tocilizumab was associated with a reduction of PWV compared to use of rituximab and abatacept (Table 3). The patients using tocilizumab also had a significant drop in BP after 3 months treatment compared to patients receiving abatacept. Reduction in inflammation and DAS28 gave a significant increase in TC and HDL-c. Changes in US scores at 3 months were associated with change in diastolic BP.

**Table 3:** Predictors of change in CVD risk markers at 3 months.

	Δ TC (β(SE)) n 32	p	Δ HDL-c (β (SE)) n 32	p	Δ LDL-c (β (SE)) n 32	p	Δ Trig (β (SE)) n 32	p	Δ PWV (β (SE)) n 35	p	Δ AIx (β (SE)) n 34	p	Δ Syst BP (β (SE)) n 35	p	Δ Diast BP (β (SE)) n 35	p
Δ CRP mg/L	-0.0 (0.0)	<0.001	-0.0 (0.0)	0.002	-0.0 (0.00)	0.02	-0.0 (0.0)	0.53	-0.0 (0.0)	0.30	-0.1 (0.1)	0.33	-0.1 (0.1)	0.07	-0.0 (0.1)	0.51
Δ ESR mm/hour	-0.0 (0.0)	<0.001	-0.0 (0.0)	0.004	-0.0 (0.0)	0.004	-0.0 (0.0)	0.57	0.0 (0.0)	0.90	-0.1 (0.1)	0.34	-0.0 (0.1)	0.79	0.0 (0.1)	0.66
Δ DAS28	-0.3 (0.1)	0.003	-0.1 (0.1)	0.01	-0.2 (0.1)	0.17	-0.1 (0.1)	0.28	0.1 (0.2)	0.72	1.0 (1.0)	0.32	3.4 (1.8)	0.07	2.1 (1.0)	0.06
Δ USBM	-0.1 (0.0)	0.01	-0.0 (0.0)	0.59	-0.0 (0.0)	0.02	-0.0 (0.0)	0.56	-0.0 (0.0)	0.29	0.1 (0.2)	0.55	0.4 (0.3)	0.17	0.4 (0.2)	0.03
Δ USPD	-0.0 (0.0)	0.13	-0.0 (0.0)	0.73	-0.3 (0.0)	0.17	-0.0 (0.0)	0.50	0.0 (0.0)	0.61	-0.1 (0.2)	0.76	0.6 (0.3)	0.06	0.5 (0.2)	0.04
Rit vs Toc	-0.2 (0.3)	0.49	-0.1 (0.2)	0.60	-0.1 (0.3)	0.76	-0.1 (0.3)	0.85	0.9 (0.4)	0.02	0.8 (3.2)	0.80	6.3 (5.0)	0.22	4.0 (2.9)	0.18
Rit vs Aba	0.4 (0.4)	0.30	-0.2(0.2)	0.37	0.6 (0.3)	0.05	0.0 (0.3)	0.88	-0.2 (0.5)	0.60	-0.7 (3.7)	0.85	-8.8 (5.8)	0.14	-6.6 (3.3)	0.06

Linear regression Dependent variables: Change (Δ) in CVD risk markers at 3 months (Baseline values subtracted from 3-months values). Independent variables: Medication and change in disease activity at 3 months (Baseline values subtracted from 3-months values). β (SE) Regression co-efficient (Standard Error), n number, CRP: C-reactive protein mg/L ESR: Erythrocyte sedimentation rate mm/hour, TC: Total cholesterol mmol/L, HDL-c: High density lipoprotein cholesterol mmol/L, LDL-c: Low density lipoprotein cholesterol mmol/L, Trig: triglycerides, DAS28: Disease activity score 28 joints, USBM: Ultrasonography B-mode score, USPD: Ultrasonography Power doppler score, Syst BP: Systolic blood pressure mmHg, Diast BP: Diastolic mmHg blood pressure, AIx: Augmentation index %, PWV: Pulse wave velocity m/s, Rit: Rituximab, Toc: Tocilizumab, Aba: Abatacept.

In the repeated analysis there were significant reductions in CRP, DAS28 and PWV over time in the rituximab group (Table 4), and a close to statistically significant decrease in BP. The early increase in lipoproteins tapered off and there were no significant changes in TC, HDL-c or TC/HDL-c over the 12-month period of rituximab use (TC/HDL-c data not shown).

**Table 4:** Estimated marginal means from mixed model linear regression for patients using Rituximab.

Variable	T1	T2	T3	T4	T1vs T2	T1vs T3	T1vs T4	T2vs T3	T2 vs T4	T3vs T4
	EM (CI)	EM (CI)	EM (CI)	EM (CI)	p	p	p	p	p	p
ESR	29.5 (21.8–37.3)	27.3 (19.5–35.1)	23.7 (15.3–32.1)	19.2 (10.7–27.7)	0.23	0.07	0.002	0.25	0.02	0.21
CRP	11.3 (4.5–18.2)	17.1 (10.2–23.9)	8.6 (0.4–16.9)	8.0 (-0.5–16.6)	0.36	0.02	0.02	0.09	0.09	0.97
DAS28	4.6 (3.4–5.7)	3.8 (2.6–5.0)	4.5 (3.2–5.7)	3.6 (2.2–5.0)	0.27	0.92	0.23	0.33	0.83	0.28
TC	5.9 (5.4–6.3)	6.0 (5.5–6.4)	6.0 (5.5–6.5)	5.6 (5.1–61)	0.49	0.55	0.17	0.99	0.05	0.07
HDL-c	1.7 (1.5–1.9)	1.7 (1.5–1.9)	1.6 (1.4–1.9)	1.7 (1.4–1.9)	0.63	0.27	0.32	0.50	0.56	0.92
LDL-c	3.6 (3.2–4.0)	3.8 (3.4–4.2)	3.8 (3.3–4.2)	3.3 (2.9–3.8)	0.36	0.49	0.14	0.05	0.02	0.05
Triglycerides	1.2 (1.0–1.5)	1.3 (1.0–1.6)	1.4 (1.1–1.8)	1.5 (1.1–1.8)	0.76	0.21	0.18	0.40	0.28	0.84
Systolic BP	127.8 (121.8–133.8)	127.5 (121.4–133.7)	123.0 (116.4–129.6)	124.2 (117.0–131.3)	0.91	0.05	0.21	0.07	0.26	0.70
Diastolic BP	79.6 (76.7–82.6)	78.9 (75.9–82.0)	77.2 (73.8–80.5)	78.4 (74.2–82.6)	0.60	0.10	0.55	0.24	0.79	0.57
PWV	7.7 (7.2–8.2)	7.7 (7.3–8.2)	7.4 (6.9–7.9)	7.3 (6.8–7.9)	0.79	0.11	0.04	0.07	0.02	0.64
AIx	22.9 (19.1–26.8)	24.4 (20.5–28.3)	25.2 (20.9–29.5)	23.6 (19.2–27.9)	0.30	0.19	0.71	0.65	0.64	0.40

Estimated marginal means (EM) after adjustment for age and gender during 1 year of treatment with Rituximab. EM: Estimated marginal means, P-values for comparison from mixed model regressions. *Result after lntransformationCRP: C-reactive protein mg/L ESR: Erythrocyte sedimentation rate mm/hour, TC: Total cholesterol mmol/L, HDL-c: High density lipoprotein cholesterol mmol/L, LDL-c: Low density lipoprotein cholesterol mmol/L, DAS28: Disease activity score 28 joints, USBM: Ultrasonography B-mode score, USPD: Ultrasonography Power doppler score, Syst BP: Systolic blood pressure mmHg, Diast BP: Diastolic mmHg blood pressure, AIx: Augmentation index %, PWV: Pulse wave velocity m/s, Rit: Rituximab, Toc: Tocilizumab, Aba: Abatacept.

## Discussion

We have reported on a reduction in PWV in patients receiving tocilizumab for 3 months. Patients receiving rituximab did not show any significant change in CVD risk markers at 3 months. However, over the 12- month period there was a significant trend of PWV reduction in patients receiving rituximab. This is the first study which has examined change of CVD risk markers following rituximab, abatacept and tocilizumab use, and the first which has found a reduction in PWV over 12 months of rituximab treatment.

The majority of studies on patients with RA receiving TNFα inhibitors have found reductions in PWV levels, fewer have reported a fall in AIx [2,11]. AIx was not significantly reduced by any of the biological DMARDs in the present study. Kume et al however, found a reduction in AIx after 6 months of tocilizumab treatment, while PWV was not assessed [11]. PWV and AIx are markers of CVD risk and both have been shown to be increased in patients with active RA [10]. But whereas PWV is a measure of arterial stiffening in central arteries, the AIx is an estimate of the augmentation of central arterial pressure caused by peripheral wave reflection and can thus show a paradoxical drop in response to increased inflammation, probably due to increase peripheral vasodilation [12].

Protogerou et al. reported an attenuation of PWV and an improved flow mediated dilatation [FMD] after 3 and 6 months on tocilizumab treatment in 11 patients with RA. Treatment with tocilizumab also gave a reduction in ESR, CRP and DAS28 in the presented study, but changes in these variables were not associated with change in PWV. Surprisingly, Mathieu found that PWV had increased despite a reduction of disease activity after 6 months of abatacept treatment in 21 patients with RA [13]. Others have also reported a lack of association between change in disease activity and change in CVD risk after treatment with TNFα inhibitors [2].

PWV was reduced by an average of 0.46 m/s during one year of rituximab treatment. This is approximately equal to 10 years of age reduction in a population of healthy young adults [14]. Mathieu et al. found change in neither PWV nor AIx during 1 year of rituximab treatment [15], the CRP level achieved after 12 months of treatment is however twice that reached in our study. Gonzalez- Juantey and Kerekes both found a significant increased endothelial function, and thus reduced CVD risk, in response to rituximab [16–17].

In analyses of the entire cohort including patients from the three treatments groups, we found that change in CRP, ESR and DAS28 at 3 months, but not type of biological DMARD received, were predictors of increased lipid levels. Increased TC and HDL-c have been found following treatment with abatacept, tocilizumab and rituximab [11,13]. Several of these studies however find that the increase in HDL-c is larger than the increase in TC, giving a net improvement in TC/HDL-c and thus an improved CVD risk profile. We did not find significant changes in TC/HDL-c at 3 or 12 months (data not shown).

USBM was associated with increased TC and reduced diastolic BP after 3 months of treatment. USBM is closely related to disease activity and inflammation and these findings were thus in accordance with the main results in this study [18]. The impact of US findings on CVD risk in RA should be studied further.

Our cohort was small and non-randomised but patients had comparable age and disease activity at baseline. Small cohorts increase the likelihood for type-II errors and the lack of significant findings in the abatacept group should be interpreted with caution. The study did not have a control group which is also a weakness. The strengths of this study were that all assessments were performed in the same environment and by experienced examiners.

In conclusion, patients with RA had an improved PWV after 3 months of treatment with tocilizumab, and 12 months of rituximab use. Disease activity and inflammation were reduced in the tocilizumab and rituximab groups and change in these parameters were negatively correlated to TC and HDL-c levels.

## Supporting Information

S1 Dataset A anonymized minimal dataset is available as supporting information. (XLS)

## References

[1] Zhang J, Chen L, Deelzell E, Munther P, Hillegass W.B, Safford M, et al The association between inflammatory markers, serum lipids and the risk of cardiovascular events in patients with rheumatoid arthritis. Annals of the Rheumatic Diseases 2014; 73 (7):1301–1308. 10.1136/annrheumdis-2013-204715 24796336

[2] Wong M, Oakley SP, Young L, Jiang B, Wierzbicki AS, Panayi G, et al Infliximab improves vascular stiffness in patients with rheumatoid arthritis. Ann Rheum Dis 2009;68:1277–84 10.1136/ard.2007.086157 18930987 PMC2703705

[3] Angel K, Provan SA, Fagerhol MK, Mowinckel P, Kvien TK, Atar D. Effect of 1-year anti-TNF-alpha therapy on aortic stiffness, carotid atherosclerosis, and calprotectin in inflammatory arthropathies: a controlled study. Am J Hypertens 2012;25(6):644–50. 10.1038/ajh.2012.12 22378036 PMC3635528

[4] Semb AG, Kvien TK, Aastveit AH, Jungner I, Pedersen TR, Walldius G, et al Lipids, myocardial infarction and ischaemic stroke in patients with rheumatoid arthritis in the Apolipoprotein-related Mortality RISk (AMORIS) Study. Ann Rheum Dis 2010; 69:1996–2001. 10.1136/ard.2009.126128 20551156

[5] Cohen SB, Emery P, Greenwald MW, Dougados M, Furie RA, Genovese MC, et al Rituximab for rheumatoid arthritis refractory to anti-tumor necrosis factor therapy: Results of a multicenter, randomized, double-blind, placebo-controlled, phase III trial evaluating primary efficacy and safety at twenty-four weeks. Arthritis Rheum 2006;54(9):2793–806. 16947627 10.1002/art.22025

[6] Kremer JM, Dougados M, Emery P, Durez P, Sibilia J, Shergy W, et al Treatment of rheumatoid arthritis with the selective costimulation modulator abatacept: twelve-month results of a phase iib, double-blind, randomized, placebo-controlled trial. Arthritis Rheum 2005;52(8):2263–71. 16052582 10.1002/art.21201

[7] Nishimoto N, Yoshizaki K, Miyasaka N, Yamamoto K, Kawai S, Takeuchi T, et al Treatment of rheumatoid arthritis with humanized anti-interleukin-6 receptor antibody: a multicenter, double-blind, placebo-controlled trial. Arthritis Rheum 2004;50(6):1761–9. 15188351 10.1002/art.20303

[8] Fransen J, Creemers MC, van Riel PL. Remission in rheumatoid arthritis: agreement of the disease activity score (DAS28) with the ARA preliminary remission criteria. Rheumatology (Oxford) 2004;43(10):1252–5. 15238643 10.1093/rheumatology/keh297

[9] Backhaus M, Burmester GR, Gerber T, Grassi W, Machold KP, Swen WA, et al Guidelines for musculoskeletal ultrasound in rheumatology. Ann Rheum Dis 2001; 60(7):641–9. 11406516 10.1136/ard.60.7.641 PMC1753749

[10] Provan SA, Semb AG, Hisdal J, Stranden E, Agewall S, Dagfinrud H, et al Remission is the goal for cardiovascular risk management in patients with rheumatoid arthritis: a cross-sectional comparative study. Ann Rheum Dis 2011;70(5):812–7. 10.1136/ard.2010.141523 21288959

[11] Kume K, Amano K, Yamada S, Hatta K, Ohta H, Kuwaba N. Tocilizumab monotherapy reduces arterial stiffness as effectively as etanercept or adalimumab monotherapy in rheumatoid arthritis: an open-label randomized controlled trial. J Rheumatol 2011;38(10):2169–71. 10.3899/jrheum.110340 21807781

[12] Vlachopoulos C, Dima I, Aznaouridis K, Vasiliadou C, Ioakeimidis N, Aggeli C, et al Acute systemic inflammation increases arterial stiffness and decreases wave reflections in healthy individuals. Circulation 2005;112(14):2193–200. 16186422 10.1161/CIRCULATIONAHA.105.535435

[13] Mathieu S, Couderc M, Glace B, Pereira B, Tournadre A, Dubost JJ, et al Effects of 6 months of abatacept treatment on aortic stiffness in patients with rheumatoid arthritis. Biologics 2013;7:259–64. 10.2147/BTT.S52003 24324327 PMC3854916

[14] McEniery CM, Yasmin, Hall IR, Qasem A, Wilkinson IB, Cockcroft JR. Normal vascular aging: differential effects on wave reflection and aortic pulse wave velocity: the Anglo-Cardiff Collaborative Trial (ACCT). J Am Coll Cardiol 2005;46(9):1753–60. 16256881 10.1016/j.jacc.2005.07.037

[15] Mathieu S, Pereira B, Dubost JJ, Lusson JR, Soubrier M. No significant change in arterial stiffness in RA after 6 months and 1 year of rituximab treatment. Rheumatology (Oxford) 2012;51(6):1107–11. 10.1093/rheumatology/kes006 22328565

[16] Kerekes G, Soltesz P, Der H, Veres K, Szabo Z, Vegvari A, et al Effects of rituximab treatment on endothelial dysfunction, carotid atherosclerosis, and lipid profile in rheumatoid arthritis. Clin Rheumatol 2009;28(6):705–10. 10.1007/s10067-009-1095-1 19319624

[17] Gonzalez-Juanatey C, Llorca J, Vazquez-Rodriguez TR, Diaz-Varela N, Garcia-Quiroga H, Gonzalez-Gay MA. Short-term improvement of endothelial function in rituximab-treated rheumatoid arthritis patients refractory to tumor necrosis factor alpha blocker therapy. Arthritis Rheum 2008;59(12):1821–4. 10.1002/art.24308 19035415

[18] Hammer HB, Sveinsson M, Kongtorp AK, Kvien TK. A 78-joints ultrasonographic assessment is associated with clinical assessments and is highly responsive to improvement in a longitudinal study of patients with rheumatoid arthritis starting adalimumab treatment. Ann Rheum Dis 2010;69(7):1349–51. 10.1136/ard.2009.126995 20472599

